# Adaptive Holography in Liquid Crystal Light-Valves

**DOI:** 10.3390/ma5091546

**Published:** 2012-08-27

**Authors:** Umberto Bortolozzo, Stefania Residori, Jean-Pierre Huignard

**Affiliations:** 1 INLN, Université de Nice-Sophia Antipolis, CNRS,1361 route des Lucioles, 06560 Valbonne, France; E-Mail: stefania.residori@inln.cnrs.fr (S.R.); 2 Jphopto, 20 Rue Campo Formio, 75013 Paris, France; E-Mail: jean-pierre.huignard@espci.fr (J.-P.H.)

**Keywords:** adaptive holography, liquid crystals, picometer detection

## Abstract

By performing two-wave mixing experiments in a liquid crystal light-valve, optical beam amplification is obtained as a strongly resonant process to which a narrow frequency bandwidth is associated. This property is exploited to realize adaptive holographic interferometric systems able to efficiently detect displacements as small as fraction of picometers. Pressure radiation induced deformations of a reflecting membrane are measured with the same type of system. Then, when used with complex wavefronts, like speckle fields, the LCLV-based interferometer allows to detect extremely small phase modulations. The examples shown demonstrate the potentialities of the light-valve for dynamic holography applications.

## 1. Introduction

Since its early developments in photorefractive crystals, dynamic holography has demonstrated great potentialities for applications in different fields such as image storage and processing [1], vibration analysis [2] and continuous reconstruction of interferograms [3]. After the seminal paper of Petrov on adaptive holographic interferometry (AHI) [4], the possibility of using the dynamic holographic recording technique to achieve highly precise measurements of small displacements has been demonstrated in numerous systems [5]. Since then, AHI has been proved as a useful tool for measuring small vibration amplitudes of reflecting objects [6,7,8,9]. A similar technique has been recently employed to realize acousto-optic imaging [10]. As a general rule, the physical mechanisms at the basis of the AHI rely on resonant two-wave mixing processes, whose associated narrow frequency bandwidth realizes the filtering function allowing to reject low frequency noise and environmental disturbances.

Starting from the last 30 years a lot of research has been devoted to photorefractive materials and their applications [11], while in the last decade spatial light modulators (SLM) have also attracted a great deal of attention and have emerged as important components for optical processing [12]. Indeed, SLMs are able to affect the phase modulation and the intensity of a readout beam, thus permitting the manipulation of information in the optical domain. In optically addressed SLM the control signal is provided by an optical input beam, so that optical parallelism can be fully exploited [13]. The general structure of an optically addressed SLM comprises two components: the photoreceptor and the electro-optic material, often separated by a dielectric mirror [14]. The input beam activates the photoreceptor which produces a corresponding charge field on the electro-optic material. The read light is modulated in its double pass through the electro-optic element in a retroreflective scheme. Since the readout can provide optical gain, this type of optically addressed SLM has also been called a light-valve. Historically, the photoreceptor has been a photoconductor, such as selenium or cadmium sulfide [15], amorphous silicon [16] or GaAs [17], and the electro-optic material has been a nematic liquid crystal layer, either in the parallel or twisted configuration [18], so that optically addressable SLM are also more widely known as liquid-crystal light-valves, LCLV.

An interesting type of LCLV has been realized by associating nematic liquid crystals with photorefractive crystals. The first photorefractive LCLV has been prepared by using as a photoconductor a thin monocrystalline $Bi_{12}SiO_{20}$ (BSO) crystal [19]. The BSO, well known for its photorefractive properties, is chosen for its large photoconductivity and dark resistance, while its transparency in the visible range allows the LCLV working in transmissive configurations, where the input and readout beams may in general coincide. These features make of the photorefractive LCLVs optical elements with attractive capabilities for numerous applications, as laser beam manipulation [20], coherent image amplification through dynamic holography [21], transverse pattern formation [22], optical beam amplification [23], wave-mixing [24], slow-light [25,26,27] and manipulation of singular beams [28]. Here, we will focus on two-beam coupling experiments and to the creation of dynamical holograms. By this way, adaptive holographic interferometry can be performed with the LCLV, in close analogy to what previously described with photorefractive crystals. The main advantage of the LCLV is that photoconductive and electro-optic properties are separately optimized, so that an excellent photosensitivity comes from the large photoconductivity of the BSO and a large nonlinear response comes from the high birefringence of the nematic liquid crystals. This is particularly important in view of the adaptive holography applications, since it allows decreasing considerably the required laser power. Moreover, the possibility to obtain large (lateral size of a few centimeters) BSO crystals with a high degree of spatial homogeneity and uniform dark resistance makes LCLV very attractive for operating over large areas, hence allowing interferometry with spatially extended optical beams.

The paper is organized as follows. In Section 2 we present the LCLV and its main characteristics. In Section 3 the basics of the two-wave mixing in the LCLV are reviewed. In Section 4 we present the theoretical background of adaptive holography in the LCLV and we derive the theoretical limit of the minimum detectable displacement. In Section 5 we report some representative experimental results, showing the ability to detect sub-picometer displacements of vibrating objects as well the possibility to reveal extremely small phase modulations in complex wavefronts. Finally, Section 6 are the conclusions.

## 2. The Liquid Crystal Light-Valve

**Figure 1:**
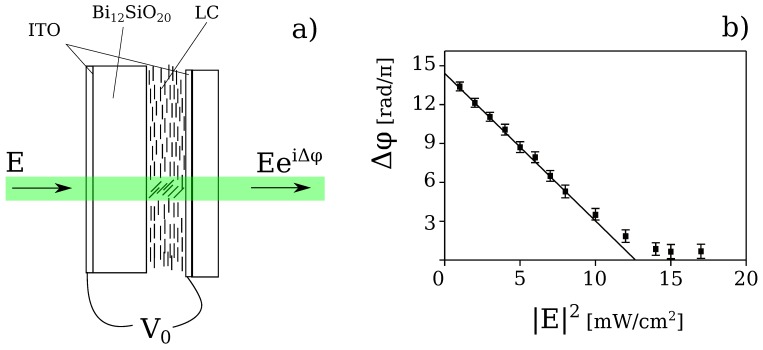
(**a**) Schematic representation of the liquid crystal light-valve; (**b**) Measured phase shift Δφ as a function of the input intensity ${\left|E\right|}^{2}$.

The liquid crystal light-valve is schematically depicted in Figure 1a. It is made by associating a liquid crystal (LC) layer with a photorefractive $Bi_{12}SiO_{20}$ (BSO) crystal, cut in the form of a thin plate (1 mm thickness, 20 × 30 $mm^{2}$ lateral size) [19,21]. The BSO is one of the confining wall and the other wall is a glass window. The thickness of the LC layer is typically of the order of 10 μm. While liquid crystals are used for their large birefringence, the BSO is used for its large photoconductivity and transparency in the visible range [11]. Transparent electrodes (*Indium Tin Oxide*, ITO, layers) deposited over the BSO and the glass wall allow the application of an external voltage $V_{0}$ across the LC layer. The voltage applied is AC, with a *rms* value from 2 to 20 *V* and a frequency from 50 Hz to 20 kHz. The liquid crystals are nematics E48 (from Merck).

The nematic phase is characterized by a long range orientational order for which all the molecules are aligned, in average, along a preferential direction, so called the nematic director ${\hat{n}}_{LC}$ [29]. Because the molecules have a different polarizability along their long and short axis, $\varepsilon_{∥}$ and $\varepsilon_{⊥}$ being the dielectric susceptibility parallel, respectively, orthogonal to the long axis of the molecules, when an electric field, or a voltage $V_{0}$, is applied across the nematic layer, an induced dipole moment arises and all the molecules reorient towards the direction of the applied field.

Because of the LC birefringence, the nematic layer as a whole behaves like a strongly birefringent material, characterized by a different refractive index for a beam polarized along the long or short molecular axis, called, respectively, the extraordinary $n_{e}$ and the ordinary $n_{o}$ index. Typical values for nematics are $n_{e}=1.7$ and $n_{o}=1.5$, which gives birefringence $\Delta n=n_{e}-n_{o}$ as large as Δn=0.2. Therefore, when the LC molecules reorient under the action of an applied field, their collective motion implies a change of the principal axis of the nematic layer, hence, an incoming light field experiences a corresponding refractive index change [30]. When a light beams impinges onto the LCLV, photo-generation of charges occurs at the BSO surface because of its photoconductive properties. Therefore, the local voltage across the LC layer increases, inducing a further molecular reorientation and thus an additional refractive index change. As a result, at the exit of the LCLV, the light beam acquires a phase shift that is a function of the applied voltage $V_{0}$ and of the total intensity of the incident beam.

A typical characteristic of the LCLV is shown in Figure 1b. The phase shift Δφ measured on the output beam is plotted as a function of the input intensity ${\left|E\right|}^{2}$, where *E* is the amplitude of the incident light beam. In the linear region of its response, the LCLV behaves as a Kerr-like nonlinear medium, providing a refractive index change proportional to the input light intensity $n=n_{0}+n_{2}{\left|E\right|}^{2}$, where $n_{0}$ is the value fixed by the applied voltage and $n_{2}$ the nonlinear coefficient. Saturation occurs when all the LC molecules are aligned along the direction of the applied electric field. Thanks to the large LC birefringence, the nonlinear coefficient, which is the slope of the linear part of the response curve, is as large as $n_{2}=-6$ $cm^{2}/W$, the minus sign accounting for the defocusing character of the nonlinearity (the refractive index changes from $n_{e}$ to $n_{o}$, with $n_{e}>n_{o}$ when LC molecules reorient under the action of the electric field).

The response time is dictated by the time $\tau_{LC}$ required by the collective motion of the LC molecules to establish over the whole thickness *d* of the nematic layer. This is given by

(1) $$\tau_{LC}∼\frac{\gamma }{K}d^{2}$$

where *γ* is the LC rotational viscosity and *K* the splay elastic constant [29]. For d=14 μm and typical values of the LC constants, $\tau_{LC}$ is of the order of 100 ms. The spatial resolution, which is the minimal size of an independently addressed area, is given by the electric coherence length of the LC

(2) $$l_{LC}∼\sqrt{\frac{\Delta \varepsilon }{K}}\frac{d}{V_{0}}$$

where $\Delta \varepsilon =\varepsilon_{∥}-\varepsilon_{⊥}$ is the dielectric anisotropy of the LC. For the usual values of $V_{0}$, $l_{LC}$ is typically of the order of 10 μm.

## 3. Two-Wave Mixing in the LCLV

**Figure 2:**
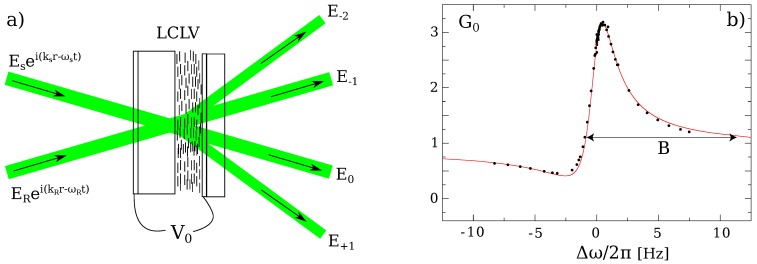
(**a**) Two-wave mixing in the liquid crystal light-valve; (**b**) Measured (points) and theoretical (line) gain $G_{0}\equiv I_{0}/I_{S}$ as a function of the frequency detuning Δω between the reference and signal beam. *B* is the frequency bandwidth characterizing the two-beam coupling process.

In the LCLV the active medium is the LC layer that is usually thin (of the order of 10 μm) when compared to the typical fringe spacing (of the order of 100 μm). Therefore, when two-wave mixing is performed in the LCLV, the beam coupling occurs in the Raman–Nath regime of diffraction. Different from the Bragg regime, for which the phase matching condition is satisfied only in one direction, the Raman–Nath diffraction produces several output order beams [31]. The interaction scheme comprises a reference beam $E_{R}$, which is sent onto the LCLV together with a signal beam $E_{S}$, as depicted in Figure 2a. The total electric field at the input of the LCLV can be written as

(3) $$E_{in}\left(\vec{r},t\right)=E_{S}e^{i[{\vec{k}}_{S}\cdot \vec{r}-\omega_{S}t]}+E_{R}e^{i[{\vec{k}}_{R}\cdot \vec{r}-\omega_{R}t]}+c.c.$$

where $E_{R}$ and $E_{S}$ are the amplitudes of the reference, respectively, the signal waves, ${\vec{k}}_{R}$ and ${\vec{k}}_{S}$ their respective propagation vectors and $\omega_{R}$, $\omega_{S}$ their frequencies. The two beams produce an intensity fringe pattern

(4) $$|E_{in}\left(\vec{r},t\right){|}^{2}=I_{T}\left[1+2\frac{E_{R}E_{S}}{I_{T}}\cos\left({\vec{K}}_{g}\cdot \vec{r}-\Delta \omega \cdot t\right)\right]$$

where $I_{T}\equiv |E_{S}{|}^{2}+{\left|E_{R}\right|}^{2}=I_{S}+I_{R}$ is the total input intensity, ${\vec{K}}_{g}={\vec{k}}_{R}-{\vec{k}}_{S}$ is the grating wave vector and $\Delta \omega =\omega_{R}-\omega_{S}$ the frequency detuning between the reference and signal. The ratio between the reference and signal intensity $\beta \equiv I_{R}/I_{S}$ is usually kept much larger than one.

The fringe pattern induces, on its turn, a photo-induced space charge distribution, hence a molecular reorientation pattern in the LC layer, which creates a refractive index grating with the same wave vector ${\vec{K}}_{g}$. The spatial period of the grating $\Lambda \equiv 2\pi /K_{g}$ is usually larger than the thickness of the LC layer. Therefore, the LC grating acts as a thin hologram, and several diffracted beams, distinguished by the numbers 0,±1,±2,...,±m, are observed at the output of the LCLV. Due to self-diffraction, photons from the pump are transferred into the different output orders. The m=0,+1,±2,... orders are amplified; that is, they receive from the reference, also called pump beam, more photons than they are losing due to the scattering on the other orders. The m=−1 order is the reference beam that, even though depleted, remains of much higher intensity than the other beams [32].

To derive the full expression for the output field, we have to consider the evolution of the amplitude $n(\vec{r},t)$ of the refractive index grating inside the LC layer. This is governed by a relaxation equation following the molecular orientation dynamics of the LC [29]

(5) $$\tau_{LC}\frac{\partial n}{\partial t}=-\left(1-l_{LC}^{2}\nabla^{2}\right)n+n_{0}+n_{2}{\left|E_{in}\right|}^{2}$$

where $l_{LC}∼10$ μm is the transverse diffusion length, $n_{0}=1.6$ is the constant value of the refractive index given by the average LC orientation under the application of the voltage $V_{0}$, and $n_{2}≃-6$ $cm^{2}/W$ is the equivalent Kerr-like coefficient of the LCLV. By coupling the above Equation (5) with the wave propagation equation for the input electric field, one can easily shows that the *m* output order field can be written as [32,33,34]

(6) $${\tilde{E}}_{m}=E_{m}e^{i({\vec{k}}_{m}\cdot \vec{r}-\omega_{m}t)}+c.c.$$

where $\omega_{m}=\omega_{S}-m\;\Delta \omega$ is the frequency, ${\vec{k}}_{m}={\vec{k}}_{S}-m\;{\vec{K}}_{g}$ the wave vector and the amplitude is given by

(7) $$E_{m}=\left[E_{S}J_{m}\left(\rho \right)+iE_{R}J_{m+1}\left(\rho \right)e^{-i\Psi }\right]\cdot e^{i\left[k\left(n_{0}+n_{2}I_{T}\right)z+m\left(\frac{\pi }{2}-\Psi \right)\right]}$$

where $J_{m}$ is the Bessel function of the first kind and of order *m*,

(8) $$\rho =\frac{2kn_{2}E_{R}E_{S}}{\sqrt{{\left(1+l_{LC}^{2}K_{g}^{2}\right)}^{2}+{\left(\Delta \omega \cdot \tau_{LC}\right)}^{2}}}d$$

is the grating amplitude and

(9) $$\tan\Psi =\frac{\Delta \omega \cdot \tau_{LC}}{1+l_{LC}^{2}K_{g}^{2}}$$

from the above expression Equation (7), we see that each order *m* receive two contributions, one is the scattering of the signal and the other is the scattering of the reference beam onto the refractive index grating.

It is useful to write each output order field in the form

(10) $${\tilde{E}}_{m}=\sqrt{G_{m}}E_{S}e^{i\Phi_{m}}e^{i({\vec{k}}_{m}\cdot \vec{r}-\omega_{m}t)}+c.c.$$

where we define $G_{m}=|E_{m}{|}^{2}/{\left|E_{S}\right|}^{2}$ as the gain factor and $\Phi_{m}$ is the associated nonlinear phase shift. Both $G_{m}$ and $\Phi_{m}$ can be calculated from Equation (7).

Let us consider the m=0 order, which coincides with the original propagation direction of the signal

(11) $${\tilde{E}}_{0}=\sqrt{G_{0}}E_{S}e^{i\Phi_{0}}e^{i({\vec{k}}_{S}\cdot \vec{r}-\omega_{S}t)}+c.c.$$

The envelope amplitude can also be written as

(12) $$E_{0}=\left[E_{S}J_{0}\left(\rho \right)+iE_{R}J_{1}\left(\rho \right)e^{-i\Psi }\right]e^{i\left[k(n_{0}+n_{2}I_{T})z\right]}$$

where $I_{T}=I_{R}+I_{S}$ is the total input intensity. The two contributions, namely the scattering of the signal and the scattering of the reference beam, sum up with their relative phases. The final effect is to produce a gain with a narrow frequency bandwidth. In Figure 2b, the gain curve $G_{0}$ is plotted as a function of the frequency detuning Δω for parameter values close to typical experimental conditions, $\tau_{LC}=120$ ms, $l_{LC}=15$ μm, Λ=150 μm, $n_{0}=1.63$, $n_{2}=-6$ $cm^{2}/W$ and β=30 and compared with the experimental data (filled circles). The maximum gain (and, correspondingly, maximum phase shift) is obtained for Δω∼0. In correspondence, a temporally modulated signal will experience a large dispersion, due to the strong selectivity of the gain whose frequency bandwidth is approximately B=10 Hz.

## 4. LCLV-Based Adaptive Holography: Setup and Theoretical Background

Adaptive holography interferometry, AHI, is realized in the LCLV by performing two-wave mixing experiments as described above. The narrow frequency bandwidth of the gain and the high nonlinear response of the LCLV, allowing its operation at low light powers, makes the LCLV an ideal medium for adaptive holography [35]. In a similar way as it occurs for AHI in photorefractive crystals [5], the gain resonance curve acts as an optical filter able to adapt the dynamic hologram by following low frequency variations and noise disturbances (inside the two-wave mixing gain bandwidth *B*).

**Figure 3:**
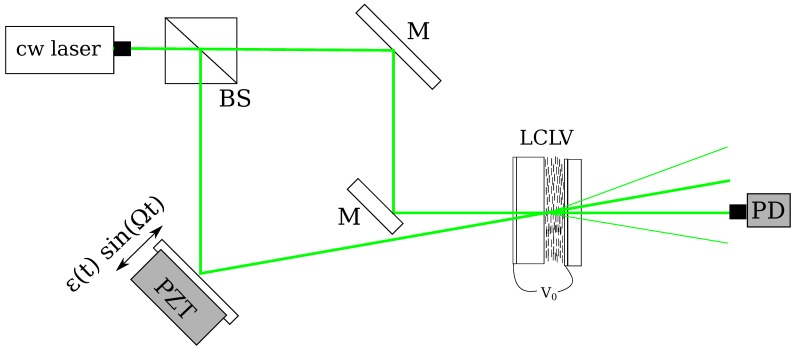
Experimental setup of the AHI interferometer with the LCLV as adaptive hologram. The input laser is split into a reference and a signal beams that are incident on the LCLV; before arriving at the LCLV the signal is sent to a vibrating object, whose amplitude of oscillation we want to detect. PZT, piezoelectrically driven mirror. The output intensity on the −1 order is measured with a photodiode PD and converted to a voltage.

The experimental setup for AHI in the LCLV is shown in Figure 3. The input beam is from a cw doubled diode-pumped solid-state laser, wavelength 532 nm, with the total input intensity typically less than 5 $mW/cm^{2}$. The laser beam is divided into a reference and a signal wave. By means of a piezoelectrically driven mirror the signal beam $E_{S}$ is phase modulated with a sinusoidal oscillation at high frequency Ω≫B and small amplitudes *ε*. The signal beam is sent onto the LCLV together with the reference beam $E_{R}$, thus producing a thin diffraction grating. Several output beams are obtained at the exit of the LCLV. The optical power of the output beams is measured with a photodiode and a lock-in amplifier.

The frequency of the modulation Ω is much greater than the bandwidth *B* of the two-wave-mixing in the LCLV. The total intensity distribution on the LCLV is

(13) $$I=|E_{R}e^{i({\vec{k}}_{R}\cdot \vec{r}+k_{0}\Delta -\omega_{0}t)}+E_{S}e^{i({\vec{k}}_{S}\cdot \vec{r}+\theta -\omega_{0}t)}{|}^{2}$$

where $\omega_{0}$ is the laser frequency, $\theta =2k_{0}\varepsilon sin\left(\Omega t\right)$ is the phase shift due to the vibrating object, ε/cosχ is the small displacement that we aim to detect, *χ* is the incidence angle of the light beam on the vibrating surface, and Δ is the optical path difference acquired by the reference and signal before arriving at the LCLV.

Given the narrow frequency bandwidth of the gain, the grating formation automatically filters out the high frequency and the amplitude of the phase grating reads as [36]

(14) $$\rho =2k_{0}dn_{2}J_{0}\left(2k_{0}\varepsilon \right)E_{R}E_{S}$$

Because of the large value of the LCLV nonlinear coefficient $n_{2}$, the process is very efficient and the output diffracted beams are easily detected. By solving the wave propagation equation in the Raman–Nath regime [35], we obtain for the optical power of the *m* output order

(15) $$P_{m}=P_{R}e^{-\alpha D}\left(K^{2}J_{m}^{2}+J_{m+1}^{2}+2KJ_{m}J_{m+1}sin\left(\theta \right)\right)$$

where α≈0.3 $cm^{-1}$ is the total absorption coefficient of the LCLV, D=1 mm the thickness of the photoconductor, $K^{2}=P_{S}/P_{R}$ the ratio between the signal and reference power and $J_{m}\equiv J_{m}\left(\rho \right)$ the Bessel function of the first kind and of order *m*. By substituting in Equation (15) the expression for *θ*, we find the component at the modulation frequency Ω

(16) $${\hat{P}}_{m}\left(\Omega \right)=4P_{R}e^{-\alpha D}KJ_{m}J_{m+1}\;J_{1}\left(2k_{0}\varepsilon \right)\sin\left(\Omega t\right)$$

where we have made use of the component parts of the Fourier–Bessel expansion [37]. Either the zero order beam, which coincides with the direction of the signal, or the −1 beam, which coincides with the direction of the reference, is detected with a photodiode and a lock-in amplifier. These orders are also used to calibrate the system. Indeed, if ρ≪1 and for m=−1 we have

(17) $${\hat{P}}_{m}\propto J_{0}\left(2k_{0}\varepsilon \right)J_{1}\left(2k_{0}\varepsilon \right)$$

which has a maximum at 1.1 rad. As already done in other AHI setups [38,39], this property has been used to find the relation between the displacement *ε* and the measured lock-in voltage $V_{lock-in}$.

### 4.1. Relative Detection Limit

For small displacements we can approximate $J_{1}\left(2k_{0}\varepsilon \right)\approx k_{0}\varepsilon$ and the detection becomes linear with *ε*, which automatically gives the highest sensitivity of the AHI interferometer. In classical interferometers, to achieve this condition the average phase difference between the interfering beams has to be set to π/2 (quadrature condition). Moreover, the AHI system does not require the stabilization with respect to variations of the optical path difference Δ, since the beam coupling is self-adapted. The sensitivity of the AHI system is obtained by considering the limit given by the photon shot-noise. The signal to noise ratio in this case can be expressed as [7,35]

(18) $$SNR=\sqrt{\frac{2\eta P_{R}}{\hbar \omega \Delta f}}e^{\frac{-\alpha D}{2}}\frac{KJ_{m}\left(\rho \right)J_{m+1}\left(\rho \right)}{\sqrt{K^{2}J_{m}^{2}\left(\rho \right)+J_{m+1}^{2}\left(\rho \right)}}2k\Delta$$

where *η* is the quantum efficiency of the photodiode and Δf is the bandwidth of the electronic detection system. The minimum detectable displacement $\varepsilon_{lim}$ is calculated by setting SNR=1. In order to compare the performances of the AHI with classical homodyne detection, the relative detection limit $\delta_{lim}^{\left(rel\right)}$ has to be considered. In the case of a classical interferometer in quadrature configuration and lossless, the ideal detection limit is [7]

(19) $$\delta_{ideal}=1/\left(2k_{0}\right){\left[\left(\hbar \omega_{0}\Delta \nu \right)/\left(2\eta P_{R}\right)\right]}^{1/2}$$

thus, we obtain

(20) $$\delta_{lim}^{\left(rel\right)}=\frac{\sqrt{K^{2}J_{m}^{2}+J_{m+1}^{2}}}{KJ_{m}J_{m+1}}e^{\frac{\alpha D}{2}}$$

the minimum $\delta_{lim}^{\left(rel\right)}\approx 1.1$ is obtained for the −1 order. Correspondingly, the maximum signal to noise ratio is obtained and the minimum detectable phase is of the order of 7 $nrad/Hz^{1/2}$.

## 5. Experimental Results: Picometer Detection

**Figure 4:**
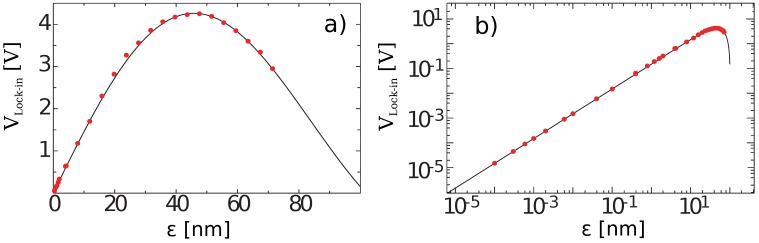
(**a**) Linear and (**b**) log scale plot of the signal $V_{lock-in}$ detected at the output of the LCLV (−1 order) *versus* the mirror displacement *ε*; frequency of modulation Ω/2π=1 kHz, $n_{2}=4.5$ $cm^{2}/W$, K=5, $P_{S}=3.2$ mW; the solid curves are the fits with the theoretical curve $J_{0}\left(2k_{0}\varepsilon \right)J_{1}\left(2k_{0}\varepsilon \right)$. From Reference [35].

The typical experimental setup for AHI with the LCLV is displayed in Figure 3. In the following set of measurements the frequency of the modulation was fixed at Ω/2π=1 kHz, which is much greater than the bandwidth B≃10 Hz of the two-wave-mixing in the LCLV. A photodiode (η≃0.63) was placed on one of the diffracted orders and the output optical power was measured by using a lock-in amplifier (1 Hz bandwidth). The signal $V_{lock-in}$ was detected at the m=−1 order, for which the theoretical curves predict the maximum sensitivity. The data are plotted in Figure 4a as a function of the mirror displacement *ε*. The intensity of the signal beam was 3 $mW/cm^{2}$ and K=5.

In Figure 4b the same data as in Figure 4a are plotted in logarithmic scale, from which it can be clearly seen that for small displacements the detection is linear. Mirror displacements as small as 0.1 pm are efficiently detected. Similar results can be obtained on the zero order. The theoretical detection limit is not reached, mainly because the smallest displacement that could be achieved was limited by the sensitivity of the vibrating system. The ultimate relative detection limit, theoretically predicted to be 1.1 times that of an ideal interferometer, could be reached in future by using more sophisticated electronics and better isolated working conditions.

### 5.1. Pressure Radiation Measurements

It is well known that the pressure *P* of normally incident cw light experienced by a macroscopic object with a plane surface has a linear dependence on the light intensity *I*

(21) $$P=\frac{I}{c}\left(1+R\right)$$

where *c* is the speed of light and *R* is the reflectivity of the illuminated surface of the object. It was demonstrated by using a photorefractive crystal that AHI is an efficient tool to measure light pressure radiation [39]. Successive experiments have demonstrated the ability of AHI to detect light radiation pressure by using the LCLV. The setup is similar to that depicted in Figure 3. A 5 μm thick nitrocellulose membrane with a reflective coating was used as a vibrating object. The membrane was placed inside a vacuum box in order to reduce air pressure perturbations. A laser beam with power 45 mW, λ=632 nm and waist 3 mm impinged on the rear side of the membrane. The laser was square modulated at a frequency 10 kHz and could be switched on/off by external control.

**Figure 5:**
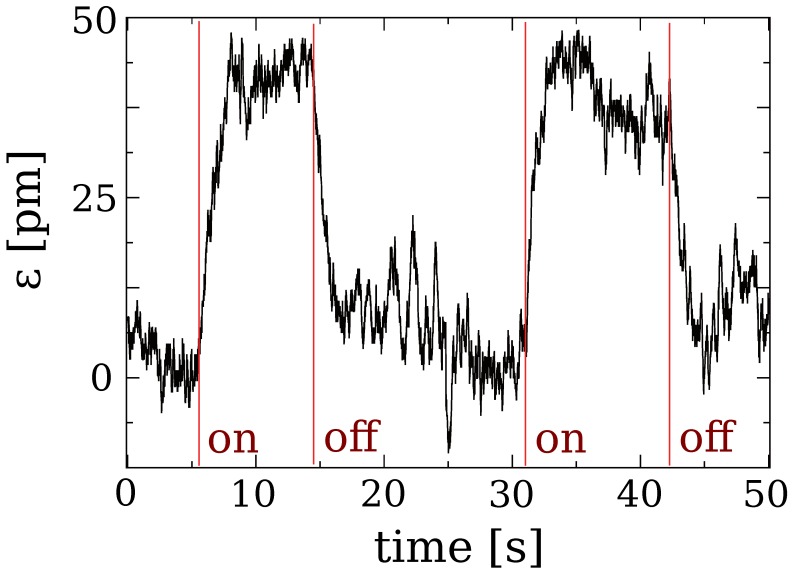
Laser-induced membrane displacement; on/off indicates the state of the laser.

The detected signal reflected by the membrane is shown in Figure 5. The laser-induced displacement can be clearly detected. The transition rate of 1 Hz is due to the bandwidth of the lock-in, whereas the noise is related to fluctuations of the membrane under the laser-induced deformation and subsequent relaxation.

### 5.2. Multimode Fiber: Acoustic Detection

**Figure 6:**
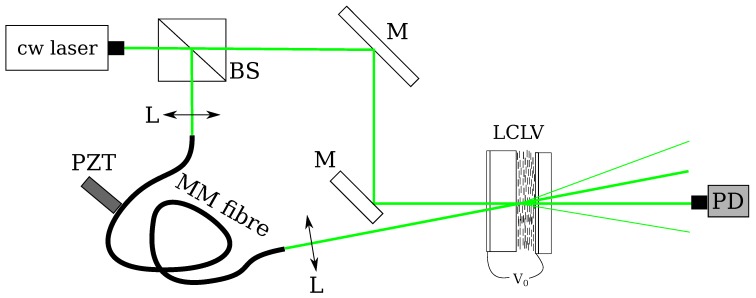
(color online). Schematic setup for the detection of phase modulations of a speckle field at the exit of a multimode fiber, MM. PZT is a piezoelectric transducer modulating in contact with the multimode fiber.

In order to test the ability of the LCLV to work with complex wavefronts, we have performed AHI by taking as signal the optical field distribution at the exit of a multimode fiber. The setup is sketched in Figure 6. A high frequency modulation is created by sending on the fiber an acoustic wave at Ω/2π=5 KHz through a piezoelectric transducer. Low frequency perturbations are induced by local, and small, disturbances, induced, for example, by touching the fiber. At the exit of the fiber, the optical field distribution has a speckle pattern with a slow dynamics.

**Figure 7:**
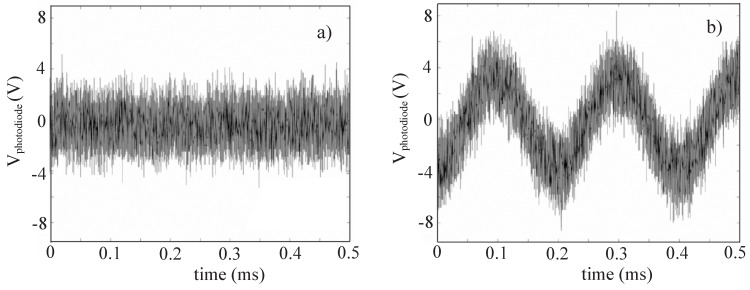
(color online). Signal $V_{photodiode}$ detected as a function of time in the case of (**a**) a Michelson interferometer and (**b**) after the LCLV in the AHI system.

In the case of a classical interferometer the phase modulations of the signal are completely hidden by the noise, as shown in Figure 7a. In the case of AHI, the two-wave mixing in the LCLV provides a narrow frequency bandwidth that filters out low frequency noise fluctuations, and the acoustic wave modulating the signal can be clearly distinguished. This is evident in Figure 7b, which plots the time evolution of the photodiode signal measured after the LCLV in the adaptive interferometer setup.

## 6. Conclusions

Nonlinear optical interactions can be efficiently implemented in liquid crystal light-valves by performing wave-mixing experiments. Optical beam amplification is obtained via two-wave mixing and a narrow frequency bandwidth is associated to the resonant character of the two-beam coupling process. This property can be exploited to realize self-adaptive interferometric systems that have been demonstrated efficient for the detection of displacements as small as fractions of picometers. Moreover, the self-adaptive character of the nonlinear process allows to perform phase detection with spatially complex wavefronts, such as speckles or distorted fields, and to detect acoustic waves in noisy environment.
